# Chronic stress exposure paradigm in zebrafish models – focusing on neurobehavioral and biochemical changes

**DOI:** 10.1192/j.eurpsy.2023.1944

**Published:** 2023-07-19

**Authors:** I. M. Balmus, R. O. Cojocariu, R. Lefter, L. Gorgan, A. Ciobica

**Affiliations:** 1 Department of Exact Sciences and Natural Sciences, Institute of Interdisciplinary Research; 2 Doctoral School of Biology, Faculty of Biology; 3 Department of Biology, Faculty of Biology, Alexandru Ioan Cuza University of Iasi; 4 Center of Biomedical Research, Romanian Academy, Iasi Branch, Iasi, Romania

## Abstract

**Introduction:**

Our previous reports on rodent models suggested that chronic stress exposure could lead to cognitive impairments, gastrointestinal transit changes, and oxidative stress. Zebrafish (*Danio rerio*) models are nowadays gaining more attention due to their advantages, as well as their complete neurobehavioral, biochemical, and genetic description and great similarity to human.

**Objectives:**

The aim of this study was to describe and to identify possible interdisciplinary applications of the neurobehavioral and biochemical changes induced by chronic stress exposure in zebrafish.

**Methods:**

The main scientific databases were screened for English-written studies describing chronic stress exposure effects in animal models. Inclusion criteria: (1) studies performed on zebrafish models; (2) reporting stress exposure effects on the animal behaviour, cognition, oxidative, and/or inflammatory status. Exclusion criteria: (1) studies not focussing on chronic stress exposure paradigm or (2) not using zebrafish models.

**Results:**

We found that chronic stress exposure was applied to larvae, juveniles, and adult zebrafish. The neurobehavioral effects were mainly suggesting memory deficits and socio-affective impairments (such as anxiety and depressive-like behaviours). Several studies found oxidative stress and inflammatory-related response in brain and gut. While our previous research experience in rodents thought us that chronic stress exposure could model functional gastrointestinal disorders of which mechanisms mainly address an impaired brain – gut interaction (irritable bowel syndrome), the data regarding the gastrointestinal status of zebrafish in similar experimental conditions are rather scarce. Some recent studies suggested that stress-exposed zebrafish showed impaired digestion and intestinal glucocorticoid receptors functions. Furthermore, it was reported that the zebrafish response to stress could be improved by probiotic administration and gut microbiota modulation.

**Conclusions:**

Chronic stress exposure paradigm is often associated with cognitive and affective-like impairments. Several oxidative stress and inflammation-related changes were also reported. Further studies are needed to describe the brain – gut interaction-associated functional gastrointestinal impairments in zebrafish.

Acknowledgements: *B. I.-M. is supported by the Project POCU/993/6/13/153322 ”Suport educațional și formativ pentru doctoranzi și tineri cercetători în pregătirea inserției în piața muncii” of the European Social Fund through the Human Capital Operational Program.

**Disclosure of Interest:**

I. M. Balmus Grant / Research support from: Project POCU/993/6/13/153322 ”Suport educațional și formativ pentru doctoranzi și tineri cercetători în pregătirea inserției în piața muncii” of the European Social Fund through the Human Capital Operational Program., R. O. Cojocariu: None Declared, R. Lefter: None Declared, L. Gorgan: None Declared, A. Ciobica: None Declared

